# Cellular and molecular regulation of the primate endometrium: a perspective

**DOI:** 10.1186/1477-7827-4-S1-S3

**Published:** 2006-10-09

**Authors:** William C Okulicz

**Affiliations:** 1 Department of Physiology, UMass Medical School, 55 Lake Avenue N., Worcester, MA 01655, USA

## Abstract

This contribution will trace some of the many seminal studies on the female uterus (endometrium) over the centuries and conclude with a description of some current research initiatives in our laboratory. Numerous contributions from many investigators over the years have contributed to our current understanding of endometrial function. The historical section of this chapter is intended to be a brief overall description of some of these efforts and not exhaustive. Additional information can be found in the review articles and books cited herein.

## Early History

For many centuries studies on the uterus were hampered by both religion and law. Early physicians were forbidden to dissect human bodies and as such their understanding of the female reproductive system was founded on their knowledge of domestic animals [1]. There is, however, some anecdotal evidence from Strabo that the ancient Egyptians (B.C.) performed ovariotomies (ovariectomies) on human females [2] although this did not apparently impact knowledge of uterine form or function. In the fourth century B.C. Greece, the Hippocratic Corpus represented in large part the beginning of modern medicine (Hippocrates, 460–377 B.C., Father of Medicine). In this work the uterus is described as a number of cavities and horns with no mention of tubes or ovaries. Only the external portions of the female reproductive tract were clearly, carefully, and accurately described. Also during this period, Aristotle described the uterus (in his writings, no drawings available) as a bicornuate structure based on his knowledge of domestic animals e.g. cow.

**Figure 1:**
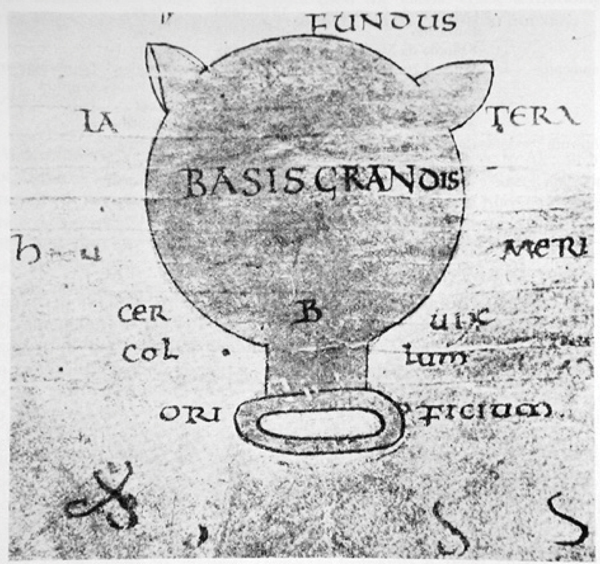
The earliest surviving illustration of uterine anatomy (9^th ^century). The drawing was based on the studies of Soranus of Ephesus [1].

Although the concept of the uterus as a bicornuate structure (Herophilus of Chalcedon and Rufus of Ephesus) continued during the early Christian era, both the uterine tubes and ovary (unknown function) were identified as separate structures. Rufus also described the "fundus" of the uterus. During this period some limited dissection of human bodies i.e. executed criminals was allowed for a limited time in Alexandria. During this same period Soranus of Ephesus incorporated his anatomical studies of the uterus along with extensive clinical discussion in his great work *Gynecology *(early second century A.D.) which influenced future physicians and investigators into the sixteenth century. Although no drawings were included in this work, a latter manuscript in the ninth century included the earliest surviving sketch of the uterus based on the studies of Soranus (Fig. 1). This representation of the uterus does contain some modern names such as fundus although Soranus supported Aristotle's view that the uterus contained cotyledons that accommodated intrauterine fetal nutrition. Soranus' work represented an advanced understanding of female reproductive anatomy, although he maintained, with his predecessors, that the ovary was a female testis. This latter concept was continued for centuries (until the seventeenth century).

Rome became the next center for medical and scientific studies on the human. Galen (130–200 A.D.) was the central figure in this effort. His contributions to uterine anatomy and function were minimal and based primarily on the previous work and opinions of Rufus and Aristotle. Of interest is that Galen did study monkeys. Galen's impact as an authority on medical knowledge and practice was enormous and influenced medicine throughout the Middle Ages. During these Dark Ages (lasting through the next millennium) medical and scientific studies on the anatomy and physiology of the uterus were scant. Knowledge in this area relied on the interpretation of past writings and previous opinions which were most often incorrect.

**Figure 2:**
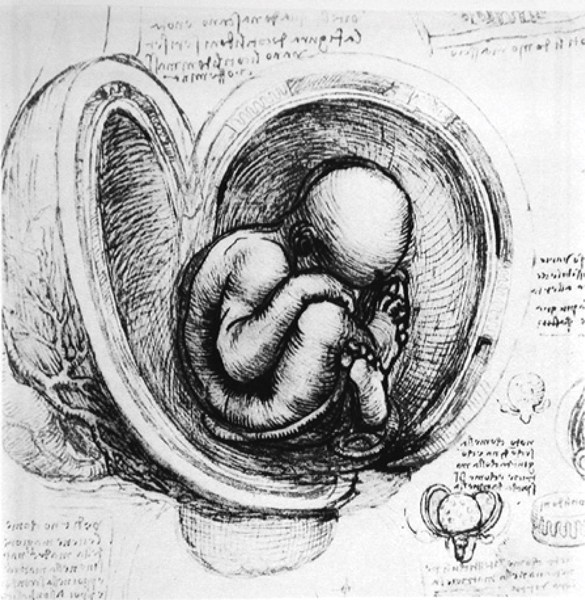
A drawing of the cavity of the uterus with fetus in situ by Leonardo da Vinci (15^th ^century). In this beautiful drawing, the rim of the placenta and a coil of the umbilical cord can be seen [1].

The 15^th ^century heralded the Renaissance and a resurgence of medical investigation guided by sound principles of scientific inquiry rather than just opinion and reinterpretation. Human dissection became accepted and the plethora of talented artists and illustrators during this period provided an outstanding resource for documentation of the anatomical characteristics of the uterus and other organs. Fig. 2, a drawing by Leonardo da Vinci that depicts an opened uterus with a fetus in situ, illustrates this marvelous connection between scientific inquiry and artistic documentation. Both the placenta and a coil of the umbilical cord can be seen in this drawing.

During this period Vesalius stood as the world's premier anatomist with his publication of *De humani corporis fabrica *in 1543. His work is widely regarded as revolutionary in anatomical science. He studied the uterus and corrected and added important modifications to its anatomy. These important observations included: it contains a single cavity; and included both muscular and decidual layers. In his treatise *Fabrica*, Vesalius first used the terms **uterus **and **pelvis**! His most famous student was Fallopio of Modena who coined the term **corpus luteum **(previously ovarian humor) and is perhaps best known for the designation, **"fallopian" tubes**.

## Recent History

The development of the compound microscope, tissue fixatives and stains coupled with access to human female reproductive tissues dramatically improved our understanding of the anatomy and cellular composition of the uterus over the next several centuries. During this period much advancement was made in the understanding that "internal secretions" and chemical mediators (messengers) played major roles in the regulation of internal organs. The hypotheses and evidence for the existence of these factors became accepted. In his Croonian lecture in 1905 at the Royal College of Physicians, "On the Chemical Correlation of the Functions of the Body", Professor Ernest H. Starling first used the word 'hormone' (from the Greek, meaning to excite or arouse) to describe these chemical messengers [2].

**Figure 3:**
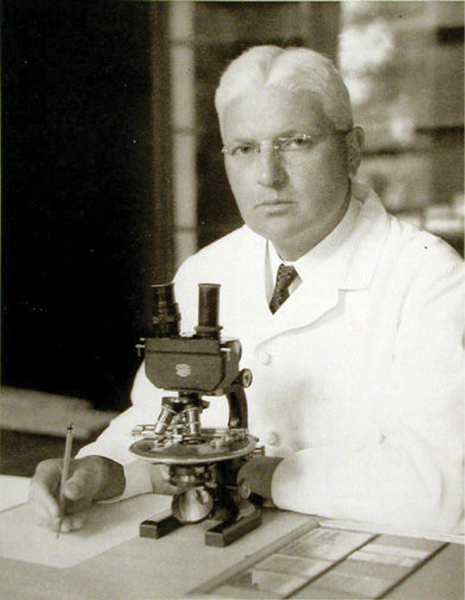
A photograph of Edgar Allen [3] who together with Adelbert Doisy identified "oestrin" (estradiol) as the hormone that promoted sexually maturity in the female rhesus monkey.

**Figure 4:**
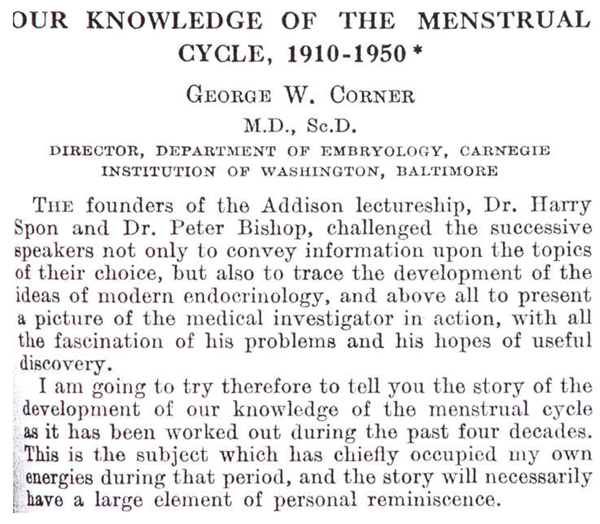
A manuscript by the brilliant scientist and humanist G.W. Corner, the discoverer of progesterone, that summarizes studies on menstruation during the first half of the 20^th ^century [10].

In the early 1920's Edgar Allen (Fig. 3) [3] and Adelbert Doisy published three papers that established the existence of the ovarian hormone "oestrin" that could induce sexual maturity in immature animals [4-6]. Allen was also able to show in the rhesus monkey (1927) that when estrogen action was stopped menstrual bleeding could be initiated [7]. These data established the concept of estrogen withdrawal as a potential mechanism in the cyclic function of the primate endometrium. During the same time (1929), Dr. George W. Corner was studying the ovulatory cycle in primates and the role of the corpora lutea. These important studies led to the discovery of the second major hormone that interacts with estrogen to affect endometrial function, namely, progesterone [8,9]. Subsequently, Corner was able to demonstrate that the decline (withdrawal) of progesterone at the end of the menstrual cycle was the critical physiological trigger for menstruation [10]. The above studies by Allen and Corner set the stage for a number of subsequent investigations on the hormonal control of the menstrual cycle and endometrial function in the 20^th ^century (Fig. 4) [10].

**Figure 5:**
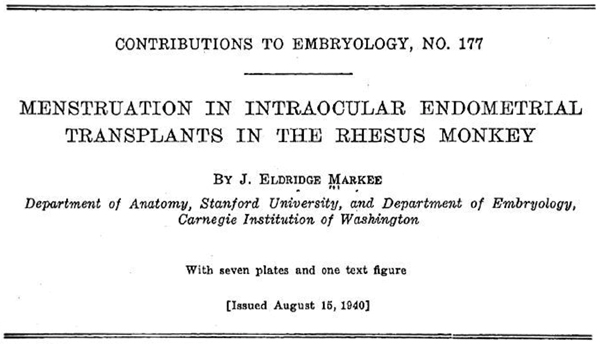
The innovative studies of Markee that used intraocular implants of endometrium in the rhesus monkey to study menstruation [11]. This work began in 1928 and was completed in 1938.

Although the above studies defined the critical hormonal elements involved in the menstrual cycle, innovative approaches were needed to study menstruation using rhesus monkey endometrium. Dr. J.E. Markee began such innovative studies in 1928 and completed these studies in 1938 (Fig. 5) [11]. The experiments involved the transplantation of endometrial pieces into the anterior chamber of the eyes of rhesus monkeys that allowed microscopic observation over numerous menstrual cycles or after various treatments in spayed animals. These studies provided a wealth of important information on a wide variety of histological parameters that set standards for thoroughness and innovation in primate endometrial research.

**Figure 6:**
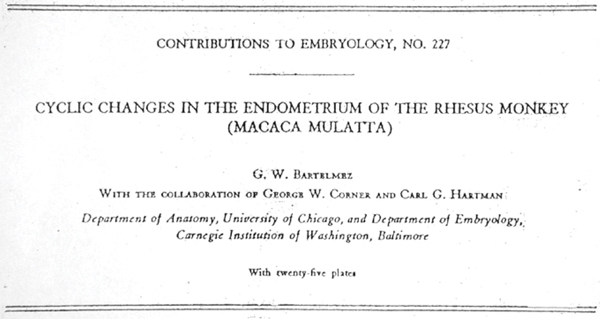
Studies by Bartelmez on the histological paramenters of the rhesus monkey throughout the menstrual cycle. [12]. These studies defined histological zones in the endometrium. Note that the collaborators include George.W. Corner and Carl G. Hartman.

Subsequent studies by Bartelmez (Fig. 6) [12]} further documented and defined zones (regions) of the rhesus endometrium using histological criteria as well as changes in these criteria during the normal menstrual cycle (note that listed collaborators included George W. Corner and Carl G. Hartman). These assiduously done studies have, and continue to serve as an important resource and reference for histological changes in the primate endometrium during the menstrual cycle including menses and regeneration. More recently the histological criteria for zonation of the primate endometrium were elaborated on by Padykula that also included transmission electron microscopy (TEM) [13,14].

**Figure 7:**
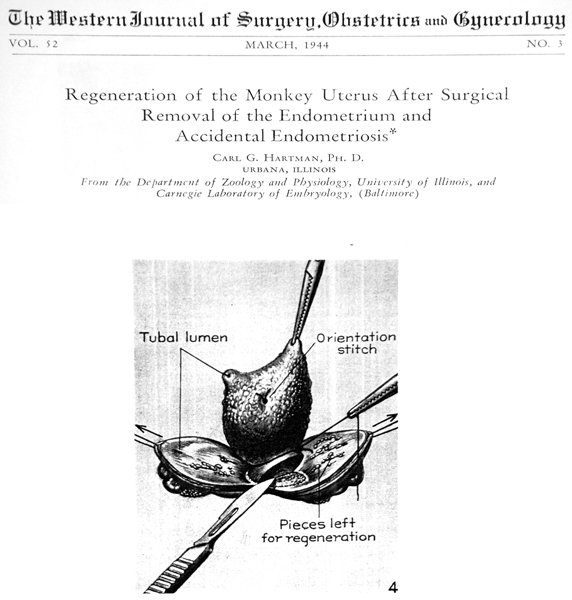
Sketch that depicts the procedure used by Hartman to study endometrial regeneration [15]. These studies demonstrated that in the absence of visible endometrial mucosa regeneration still occurred.

The regeneration of the endometrium during and following menses is clearly an important event for continued reproductive competence. The tissue components that were necessary to achieve this end result were, however, unclear. A few years after Markee's studies (above), Dr. Carl G. Hartman initiated another series of novel experiments to identify such 'regenerative components' in the rhesus monkey. After careful surgical removal of the endometrium, including both the functionalis and the basalis, he was able to show that complete regeneration still occurred (Fig. 7) [15]. Hartman's studies continue to have an important impact on our ongoing understanding of normal regeneration during menses and the cellular components/stem cells that may play roles in this process [16-18].

**Figure 8:**
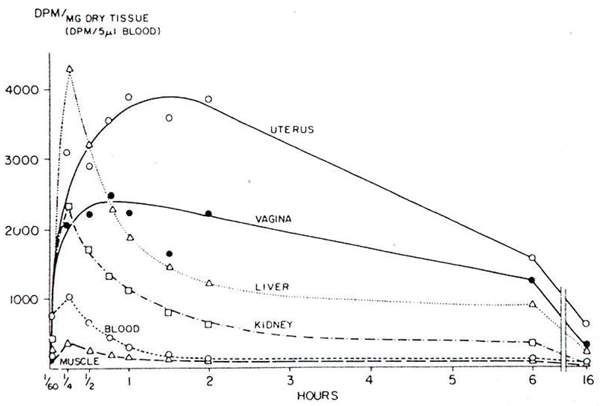
Tritiated estradiol was used as a tracer by Jensen and Jacobsen to study in vivo uptake and retention in the rat [20]. This classic study helped to establish the presence of estrogen receptors in target tissues.

**Figure 9:**
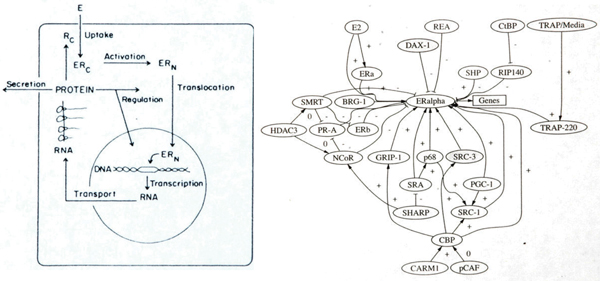
A depiction of estrogen receptor interaction almost 25yrs ago (left panel) [19] and a current view of estrogen receptor interactions (right panel) [23,25].

Although the effects of estrogen (estradiol) on the endometrium were well-known, the mechanism(s) remained unclear. A breakthrough in our understanding occurred with the synthesis of radioactive steroid that could be used for in vivo tracer studies. The first tracers to be used were tritiated hexestrol and estradiol in 1959 (Glascock and Hoekstra) and 1960 (Jensen and Jacobson) respectively [19]. Fig. 8 shows an example of the classic uptake and retention studies of Jensen and colleagues using tritiated estradiol [20]. These studies and others [21] were able to demonstrate the selective binding of estradiol in target tissues and established the means to study estrogen receptors and other steroid hormone receptors for the next several decades. The availability of radioactive steroids of high specific activity now allowed numerous studies to be done on the function and mechanism of action of both estrogen and progesterone receptors. Fig. 9 (left panel) depicts an early model of estrogen receptor action and how this transcription factor affects the cellular machinery to achieve its biochemical responses [19]. Since then, studies by many investigators have demonstrated numerous estrogen receptor interactions with other proteins that are involved in estrogen receptor alpha action (Fig. 9, right hand panel) [22-25]. This latter scheme shows only estrogen receptor alpha interactions. Recently, a second receptor, estrogen receptor beta, has also been discovered [26]. In addition, there is recent evidence to support a role for membrane receptors for estrogen that can mediate cellular responses [27,28].

**Figure 10:**
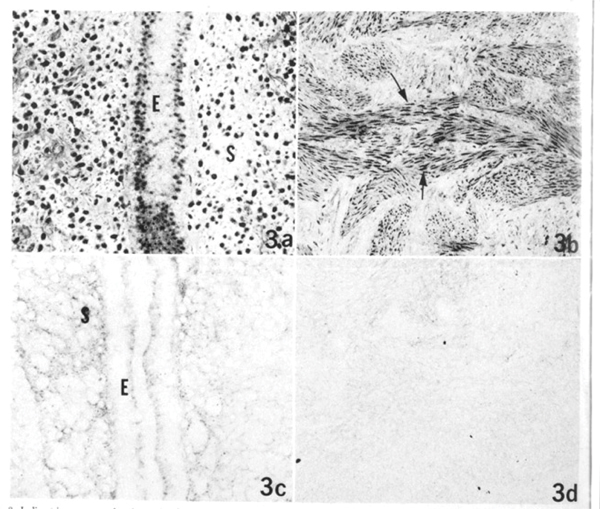
Immunohistochemical analysis of the estrogen receptor in the rhesus monkey endometrium (left panel) and myometrium (right panel) with appropriate controls below. These studies in Brenner's laboratory were the first in the rhesus monkey endometrium [31] and demonstrated that immunoreactive estrogen receptor resided primarily in the nuclear compartment.

One of the next breakthroughs in studies on hormone receptors and their regulation in the endometrium was the development of a specific antibody to the human estrogen receptor. These important studies were conducted in the laboratory of Elwood Jensen [29]. The regional and cellular complexity of the endometrium was well-known and the availability of this antibody now allowed immunohistochemical analyses of the estrogen receptor. Press [30] and Brenner [31] were the first to use this approach in the human and rhesus monkey endometrium respectively. In the rhesus monkey, estrogen receptor immunoreactivity was shown to be present in both stromal and epithelial cells throughout the endometrium and myometrium following estrogen treatment (Fig. 10) [31]. Importantly, this study, and others demonstrated that the estrogen receptor resided primarily in the nuclear compartment [30,32]. The above studies provided important evidence to alter the previously held view that the estrogen receptor resided primarily in the cytoplasm of cells and translocated to the nucleus upon hormone binding (see Fig. 9).

**Figure 11:**
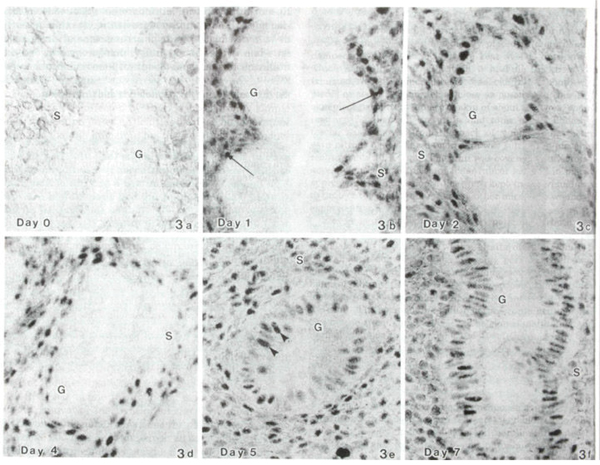
Demonstration that immunoreactive estrogen receptor was dramatically reduced by progesterone treatment in the rhesus monkey endometrium [37]. The top left panel shows the absence of estrogen receptor staining in the endometrial functionalis after estrogen plus progesterone. The other panels monitor the return of estrogen receptor immunoreactivity during progesterone withdrawal (days 1–7).

Previous studies had shown that progesterone dramatically decreased estrogen receptor binding in the uterus and endometrium [33-36]. This effect of progesterone appeared to act as an important mechanism for the modulation of estrogen action and permit a complete progestational response in the uterus. Using immunohistochemical analysis, Brenner's laboratory was able to demonstrate a dramatic decrease of immunoreactive (primarily nuclear) estrogen receptor in both stromal and epithelial cells in rhesus monkey endometrium following progesterone treatment (Fig. 11) [37]. This effect of progesterone on immunoreactive estrogen receptor in the primate endometrium was also observed in other laboratories [34,38-41].

The above immunohistochemical approaches allowed the identification of potential estrogen responsive cells versus unresponsive cells in a variety of tissues that contain heterogeneous cell populations and were not amenable to steroid binding assays. For example, this approach has been applied clinically as a diagnostic tool, specifically breast cancer biopsy specimens, to help identify estrogen responsive cells/tumors [42-44]. In the following years antibodies to the progesterone receptor became widely available and spawned a number of studies on the human and primate endometrium [36,39,41,45-47].

**Figure 12:**
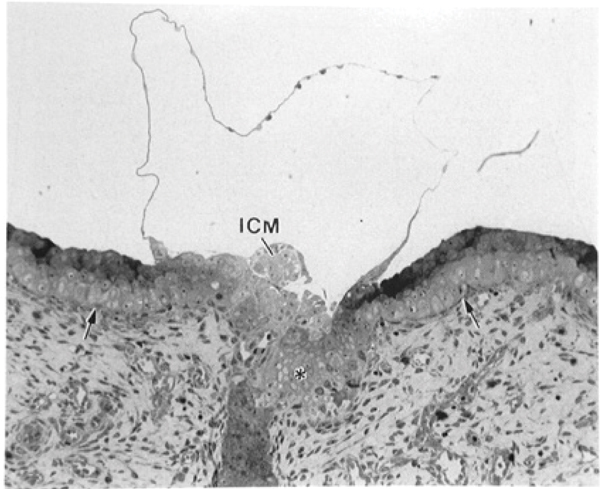
Rhesus monkey implantation site one date after implantation as described by Allen Enders [49]. ICM (inner cell mass).

In the early 1980's the role of the ovaries on endometrial receptivity for implantation in the rhesus monkey was studied by Hodgen [48]. In these studies, ovariectomized female monkeys were placed on artificial menstrual cycles using silastic implants of estradiol or progesterone. Surrogate embryo transfer was initiated during the expected window of receptivity during this protocol. Implantation occurred and hormonal support (progesterone implants) for pregnancy allowed subsequent delivery. These studies suggested that ovarian support for implantation could be mimicked with only estradiol and progesterone hormonal support. Blastocyst implantation in the primate endometrium has also been studied extensively and elegantly over the years by Allen Enders [49]. An excellent example of this work (Fig. 12) depicts the implantation site of a rhesus monkey one day after implantation. This demonstration dramatically illustrates the fundamental role of the endometrium in primate reproduction.

## New Initiatives and Approaches

There are a number of approaches that have been used in the past to describe and analyze the hormonal influences and mechanisms that govern primate endometrial responses. These have included: gross anatomy, morphology, and histology; hormonal manipulation; steroid binding assays; immunohistochemical analyses (see above). Transmission and scanning electron microscopy as well as ultrasound have also been used to study uterine (endometrial) structure [14,50-52]. A number of new and powerful cellular and molecular techniques have recently been added to our arsenal of approaches to study tissue and cell-type gene and protein expression. These include: PCR analysis of gene expression; differential display; gene microarray analyses; laser capture microdissection; and proteomic analysis. Our laboratory has recently embarked on new studies of the rhesus monkey endometrium with several of these molecular approaches and we present some of our recent work below.

**Figure 13:**
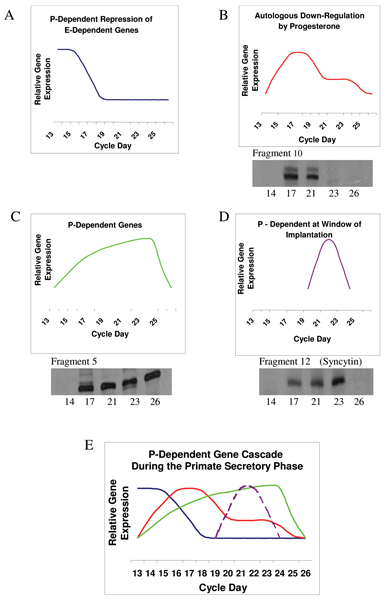
A schematic representation together with examples of relative gene expression patterns in the rhesus endometrium during the secretory phase [56]. A) progesterone (P) repression of estradiol (E)-dependent genes. B) autologous down-regulation of P-dependent genes e.g. fragment 10. C) P-induction of genes during the secretory phase e.g. fragment 5. D) P-induction of gene expression during the window of receptivity e.g. fragment 12, syncytin. E) a composite cascade of gene expression patterns A-D. The associated panel (B-D) for each fragment shows the relevant temporal portion of the gel following PCR analyses.

**Figure 14:**
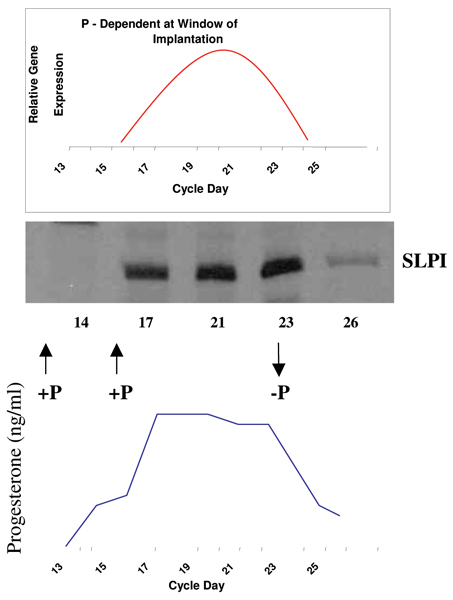
SLPI expression and hormonal levels of progesterone (P) during the expected window of receptivity in the rhesus monkey endometrium [56]. Upper panel shows the hypothetical expression pattern (red line) and the associated temporal pattern of SLPI expression as determined by PCR analyses. The lower panel shows the associated serum progesterone levels during this timeframe.

Our previously published studies [34,53-55] have described in detail the protocols for creation of both adequate and inadequate menstrual cycles in ovariectomized rhesus monkey. We chose to use the technique of differential display with cDNA populations harvested during the expected window of endometrial receptivity (days 17–26) in adequate cycles [56]. The technique of differential display allows for the identification of mRNAs that show differenes in expression level between two or more cDNA populations or that are unique to a cell type, tissue, or developmental stage [57]. Different patterns (bands) of expression will be observed following electrophoresis and autoradiography of the radioactive fragments [58]. Subsequently, the differential expression of these gene fragments can be confirmed by PCR analysis and, if appropriate, sequenced. We had previously hypothesized different patterns of gene expression during the secretory phase in the rhesus endometrium based on our work and other studies in the literature [59]. The results of our studies are shown in Fig. 13 and the hypothetical patterns of expression are described in the legend (see also panel E) [56]. Of particular interest are the fragments that displayed a restricted expression during the expected window of receptivity (panel D). Two fragments exhibited high homology to previously characterized human genes and a third fragment contained motifs of interest: 1. syncytin (shown in Figure 13, panel D, also known as endogenous retrovirus W (HERV-W) envelope protein, a highly fusogenic membrane glycoprotein that induces formation of giant syncytia and believed to be important in decidual and placental development; 2. secretory leukocyte protease inhibitor (SLPI) (Fig. 14), also known as antileukoprotease, an endometrial neutrophil elastase inhibitor with antibacterial and anti-inflamatory properties; and 3. a fragment similar to BAT2 that contains motifs typical of the integrin receptor family. We have also studied the expression of these genes during inadequate secretory phases and have shown that all three of these genes are underrepresented compared to their expression in adequate secretory phases (see below).

**Figure 15:**
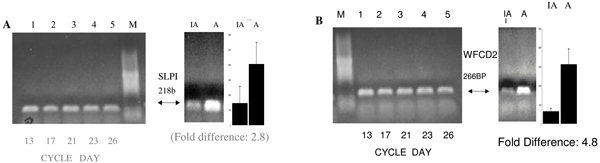
Semiquantitative PCR analysis comparing Inadequate (IA) and Adequate (A) cDNA populations on cycle days 13, 17, 21, 23, and 26 for SLPI and WFDC2 (panels A and B respectively) [66]. Lane M is a size marker (100 bp DNA ladder). The side panels compare IA and A cDNA populations on day 23 (midsecretory phase) for each gene and the column charts represent average densitometric analysis of three experiments and margin of error (SD).

**Figure 16:**
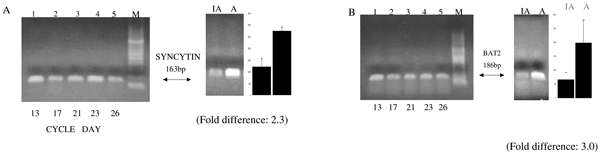
Semiquantitative PCR analyses of endometrial cDNA populations for syncytin and BAT2 (panels A and B respectively) prepared from Inadequate (IA) cycle days 13, 17, 21, 23, and 26 [66]. The side panels compare IA and Adequate (A) cDNA populations on day 23 for each gene. The column charts represent average densitometric analyses of three experiments with margins of error (SD).

We as well as other investigators [60-65] have used the powerful but often perplexing technique of gene expression (microarray) profiling to identify differentially expressed genes in the human and nonhuman primate endometrium. A complete discussion of these data is beyond the scope of this chapter. Our studies [64] were focused on the identification of progesterone-regulated endometrial genes during the rhesus monkey secretory phase. Specifically, our data confirmed the elevated expression of SLPI (see above) and identified another member of the SLPI family of secretory proteins, Whey acidic protein four-disulfide core domain 2 (WFDC2). WFDC2 showed a restricted expression similar to that of SLPI during the window of receptivity. We have also studied the temporal regulation of these two endometrial genes during inadequate secretory phases [66]. Our results clearly showed that the expression of both SLPI and WFDC2 is not temporally elevated during an inadequate secretory phase (2.8- and 4.8-fold decrease respectively) (Fig. 15). In addition, we have also shown that both syncytin and BAT2 (see above) are also temporally underrepresented during an inadequate secretory phase (2.3- and 3.0-fold decrease respectively) (Fig. 16). Together, these data suggest that these four genes may play important roles in endometrial maturation and function during the receptive period of an adequate secretory phase.

**Figure 17:**
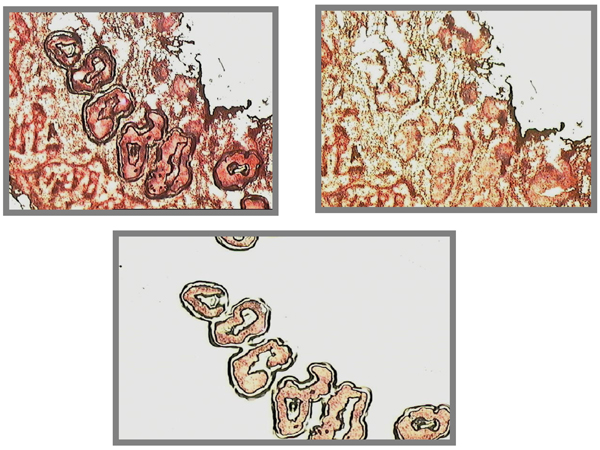
Laser capture microdissection of rhesus monkey endometrial glands [67]. Top left panel shows the laser etching of glands, top right panel shows the glands removed by the capture film, and the bottom panel shows the glands captured on the film.

We have previously used the powerful technique of laser capture microdissection (LCM) coupled with differential display to identify differentially expressed genes between cell-types (stroma versus epithelia) in different regions of the endometrium (functionalis versus basalis) during the mid-secretory phase of adequate cycles [67,68]. Fig. 17 shows an example of the removal of glandular elements from an endometrium using this technique. The top left panel shows the laser etching of the glands, the top right panel shows that the glands were removed by the capture film, and the bottom panel shows that the glands were captured on the film. Our studies showed that several gene fragments displayed a differential pattern of expression among cell-types or regions. For example, a known gene, the leukotriene B4 receptor gene, was primarily expressed in both stroma and epithelia of the functionalis. Levels of LTB4 have been shown to be elevated in dysmenorrheal and endometriosis [69]. In addition, LBT4 levels have been shown to be elevated during the peri-implantation period in the rat [70].

**Figure 18:**
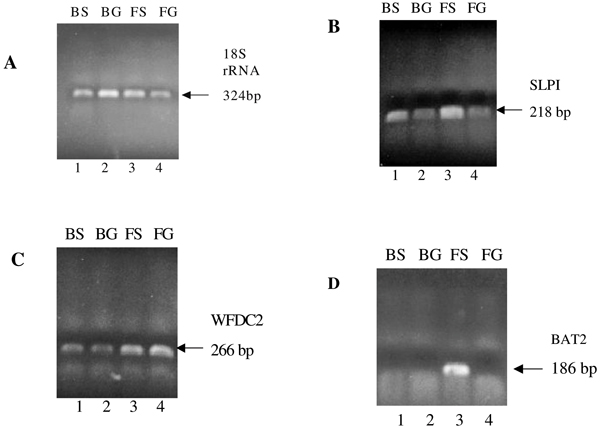
Semiquantitative PCR analysis of cDNA populations prepared from laser capture microdissected rhesus monkey endometria from adequate secretory phases [66]. Basalis stroma (BS), basalis glands (BG), functionalis stroma (FS) and functionalis glands (FG) were analyzed for 18S rRNA, SLPI, WFDC2 and BAT2 panels A, B, C and D respectively.

We have also recently used LCM to study cell-type and regional differences in expression of SLPI, WFDC2, BAT2, and syncytin (see above) in the rhesus endometrium [66]. SLPI and BAT2 showed an increased expression primarily in the stromal compartment of the functionalis whereas WFDC2 displayed elevated expression in both stroma and epithelia of the functionalis compared to the basalis (Fig. 18). No detectable differences for syncytin were observed with these LCM samples. These studies demonstrate a differential expression of several known genes during the window of receptivity that appears to be primarily localized in the functionalis of the primate endometrium, the primary target for blastocyst invasion. Further studies are necessary to understand the functions and roles of these genes in primate endometrial maturation and receptivity.

## Conclusion

This chapter has attempted to provide an historical perspective on the progress of our knowledge of the primate endometrium. This account and the early and recent historical citations echo the truism that all of us as investigators learn from and "stand on the shoulders" of those who have preceded us. Although ideas and concepts have, for the most part, driven our scientific inquiry, some "opinions" do not easily fall out of favor e.g. female testes and will continue to require our vigilant scrutiny. Some new approaches that have been developed and used to investigate the primate endometrium have also been noted. The applications of these tools and others yet to be developed will allow our ideas and hypotheses to be tested and lead us down new pathways of discovery and knowledge.

## References

[B1] Ramsey EM, Wynn RM (1977). History. Biology of the Uterus.

[B2] Medvei CM, Hingham MA (1982). A History of Endocrinology.

[B3] Young WC, Young WC, Corner GW (1961). Edgar Allen. Sex and Internal Secretions.

[B4] Allen E, Doisy EA (1924). The extraction and some properties of an ovarian hormone. J Biol Chem.

[B5] Allen E, Doisy EA (1924). The induction of a sexually mature condition in immature females by injection of the ovarian follicular hormone. Am J Physiol.

[B6] Allen E, Doisy EA (1923). An ovarian hormone. J Am Med Assoc.

[B7] Allen E (1927). The menstrual cycle of the monkey, macacus rhesus observations on normal animals, the effects of removal of the ovaries and the effects of injections of ovarian and placental extracts into the spayed animals. Contrib Embryol Carnegie Inst.

[B8] Corner GW, Allen W, Myron W (1929). Physiology of the corpus luteum (II). Production of a special reaction progestational proliferation, by extracts of the corpus luteum. Am J Physiol.

[B9] Corner GW, Allen W, Myron W (1929). Normal growth and implantation of embryos after very early ablation of the ovaries, under the influence of extracts of the corpus luteum. Am J Physiol.

[B10] Corner GW (1951). Our knowledge of the menstrual cycle, 1910–1950. Lancet.

[B11] Markee JE (1940). Menstruation in intraocular endometrial explants in the rhesus monkey. Contrib Embryol Carnegie Inst.

[B12] Bartelmez GW, Corner GW, Hartman CG (1951). Cyclic changes in the endometrium of the rhesus monkey (Macaca mulatta). Contrib Embryol.

[B13] Padykula HA, Coles LG, Okulicz WC, Rapaport SI, McCracken JA, King NW, Longcope C, Kaiserman-Abramof IR (1989). The basalis of the primate endometrium: A bifunctional germinal compartment. Biol Reprod.

[B14] Padykula HA, Coles LG, McCracken JA, King NW, Longcope C, Kaiserman-Abramof IR (1984). A zonal pattern of cell proliferation and differentiation in the rhesus endometrium during the estrogen surge. Biol Reprod.

[B15] Hartman CG (1944). Regeneration of the monkey uterus after surgical removal of endometrium and accidental endometriosis. W J Surg Obstet Gynecol.

[B16] Padykula HA (1991). Regeneration in the primate uterus: The role of stem cells. Ann NY Acad Sci.

[B17] Ferenczy A, Fenoglio CM, Wolff M (1980). Regeneration of the human endometrium. Progress in Surgical Pathology.

[B18] Okulicz WC, Glasser SR, Aplin JD, Giudice L, Tabibzadeh S (2002). Regeneration. The Endometrium.

[B19] Jensen EV, Greene GL, Closs LE, DeSombre ER, Nadji M, Nadji M (1982). Receptors Reconsidered: A 20-year Perspective. Recent Prog Horm Res.

[B20] Jensen EV, Jacobson HI, Pincus G, Vollmer EP (1960). Fate of steroid estrogens in target tissue. Biological Activities of Steroids in Relation to Cancer.

[B21] Gorski J (1994). A hindsight view of early studies on the estrogen receptor: A personal history. Steroids.

[B22] McKenna NJ, Lanz RB, O'Malley BW (1999). Nuclear receptor coregulators: Cellular and molecular biology. Endocr Rev.

[B23] McDonnell DP, Norris JD (2002). Connections and regulation of the human estrogen receptor. Science.

[B24] O'Malley BW (2005). A life-long search for the molecular pathways of steroid hormone action. Mol Endocrinol.

[B25] Norris JD, McDonnell DP (2005). Estrogen receptor pathway. Sci Stke Connection Map.

[B26] Zhang WH, Andersson S, Cheng GJ, Simpson ER, Warner M, Gustafsson JÅ (2003). Update on estrogen signaling. FEBS Lett.

[B27] Watson CS, Campbell CH, Gametchu B (2002). The dynamic and elusive membrane estrogen receptor-α. Steroids.

[B28] Walters MR, Nemere I (2004). Receptors for steroid hormones: membrane-associated and nuclear forms. Cell Mol Life Sci.

[B29] Greene GL, Fitch FW, Jensen EV (1980). Monoclonal antibodies to estrophilin. Probes for the study of estrogen receptors. Proc Natl Acad Sci USA.

[B30] Press MF, Nousek-Goebl N, King WJ, Herbst AL, Greene GL (1984). Immunohistochemical assessment of estrogen receptor distribution in the human endometrium throughout the menstrual cycle. Lab Invest.

[B31] McClellan MC, West NB, Tacha DE, Greene GL, Brenner RM (1984). Immunocytochemical localization of estrogen receptors in the macaque reproductive tract with monoclonal antiestrophilins. Endocrinology.

[B32] Welshons WV, Lieberman ME, Gorski J (1984). Nuclear localization of unoccupied oestrogen receptors. Nature.

[B33] West NB, Brenner RM (1985). Progesterone-mediated suppression of estradiol receptors in cynomolgus macaque cervix endometrium, and oviduct during sequential estradiol-progesterone treatment. J Steroid Biochem.

[B34] Okulicz WC, Savasta AM, Hoberg LM, Longcope C (1990). Biochemical and immunohistochemical analyses of estrogen and progesterone receptors in the rhesus monkey uterus during the proliferative and secretory phases of artificial menstrual cycles. Fertil Steril.

[B35] Clark JH, Peck EJ (1979). Female Sex Steroids Receptors and Function.

[B36] Okulicz WC, Hild-Petito S, Chilton B, Bazer FW, Totowas, NJ (1998). Expression of Steroid Hormone Receptors in the Pregnant Uterus. The Endocrinology of Pregnancy.

[B37] McClellan MC, West NB, Brenner RM (1986). Immunocytochemical localization of estrogen receptors in the macaque endometrium during the luteal-follicular transition. Endocrinology.

[B38] Lessey BA, Killam AP, Metzger DA, Haney AF, Greene GL, McCarty KS (1988). Immunohistochemical analysis of human uterine estrogen and progesterone receptors throughout the menstrual cycle. J Clin Endocrinol Metab.

[B39] Hild-Petito S, Verhage HG, Fazleabas AT (1992). Immunocytochemical localization of estrogen and progestin receptors in the baboon (*Papio anubis*) uterus during implantation and pregnancy. Endocrinology.

[B40] Okulicz WC, Balsamo M, Tast J (1993). Progesterone regulation of endometrial estrogen receptor and cell proliferation during the late proliferative and secretory phase in artificial menstrual cycles in the rhesus monkey. Biol Reprod.

[B41] Lessey BA, Yeh IT, Castelbaum AJ, Fritz MA, Ilesanmi AO, Korzeniowski P, Sun JH, Chwalisz K (1996). Endometrial progesterone receptors and markers of uterine receptivity in the window of implantation. Fertil Steril.

[B42] Barnes DM, Harris WH, Smith P, Millis RR, Rubens RD (1996). Immunohistochemical determination of oestrogen receptor: Comparison of different methods of assessment of staining and correlation with clinical outcome of breast cancer patients. Br J Cancer.

[B43] Harvey JM, Clark GM, Osborne CK, Allred DC (1999). Estrogen receptor status by immunohistochemistry is superior to the ligand-binding assay for predicting response to adjuvant endocrine therapy in breast cancer. J Clin Oncol.

[B44] Leake R, Barnes D, Pinder S, Ellis I, Anderson L, Anderson T, Adamson R, Rhodes T, Miller I, Walker R, UK RG, UK NEQA, Scottish Breast Canc Pathol Grp, Receptor Biomarker Study Grp EORTC (2000). Immunohistochemical detection of steroid receptors in breast cancer: a working protocol. J Clin Pathol.

[B45] Press MF, Udove JA, Greene GL (1988). Progesterone receptor distribution in the human endometrium. Am J Pathol.

[B46] Brenner RM, McClellan MC, West NB, Novy MJ, Haluska GJ, Sternfeld MD (1991). Estrogen and progestin receptors in the macaque endometrium. Ann NY Acad Sci.

[B47] Okulicz WC, Scarrell R (1998). Estrogen receptor alpha and progesterone receptor (A/B) in the rhesus endometrium during the late secretory phase and menses. Proc Soc Exp Biol Med.

[B48] Hodgen GD (1983). Surrogate embryo transfer combined with estrogen-progesterone therapy in monkeys. Implantation, gestation, and delivery without ovaries. JAMA.

[B49] Enders AC, Glasser SAJGLTS (2002). Blastocyst II. Implantation in Primates. The Endometrium.

[B50] Blankenship TN, Enders AC (2003). Modification of uterine vasculature during pregnancy in macaques. Microsc Res Tech.

[B51] Bhartiya D, Bajpai VK (1995). Cyclic alterations in rhesus monkey endometrium by scanning electron microscopy. Reprod Fertil Dev.

[B52] Morgan PM, Hutz RJ, Kraus EM, Bavister BD (1987). Ultrasonographic assessment of the endometrium in rhesus monkeys during the normal menstrual cycle. Biol Reprod.

[B53] Longcope C, Bourget C, Meciak PA, Okulicz WC, McCracken JA, Hoberg LM, Padykula HA (1988). Estrogen dynamics in the female rhesus monkey. Biol Reprod.

[B54] Okulicz WC, Balsamo M, Tast J (1993). Progesterone regulation of endometrial estrogen receptor and proliferation during the late proliferative and secretory phase in artificial menstrual cycles in the rhesus monkey. Biol Reprod.

[B55] Okulicz WC, Ace CI, Longcope C, Tast J (1996). Analysis of differential gene regulation in adequate *versus *inadequate secretory-phase endometrial complementary deoxyribonucleic acid populations from the rhesus monkey. Endocrinology.

[B56] Okulicz WC, Ace CI (2003). Temporal regulation of gene expression during the expected window of receptivity in the rhesus monkey endometrium. Biol Reprod.

[B57] Liang P, Pardee A (1992). Differential display of eukaryotic messenger RNA by means of polymerase chain reaction. Science.

[B58] Ace CI, Okulicz WC (1999). Identification of progesterone-dependent mRNA regulatory patterns in the rhesus monkey endometrium by differential display reverse-transcriptase-polymerase chain reaction. Biol Reprod.

[B59] Okulicz WC, Ace CI (1999). Progesterone-regulated gene expression in the primate endometrium. Semin Reprod Endocrinol.

[B60] Kao LC, Tulac S, Lobo S, Imani B, Yang JP, Germeyer A, Osteen K, Taylor RN, Lessey BA, Giudice LC (2002). Global gene profiling in human endometrium during the window of implantation. Endocrinology.

[B61] Carson DD, Lagow E, Thathiah A, Al-Shami R, Farach-Carson MC, Vernon M, Yuan L, Fritz MA, Lessey B (2002). Changes in gene expression during the early to mid-luteal (receptive phase) transition in human endometrium detected by high-density microarray screening. Mol Hum Reprod.

[B62] Borthwick JM, Charnock-Jones DS, Tom BD, Hull ML, Teirney R, Phillops SC, Smith SK (2003). Determination of the transcript profile of human endometrium. Mol Hum Reprod.

[B63] Riesewijk A, Martin J, van Os R, Horcajadas JA, Polman J, Pellicer A, Mosselman S, Simon C (2003). Gene expression profiling of human endometrial receptivity on days LH+2 versus LH+7 by microarray technology. Mol Hum Reprod.

[B64] Ace CI, Okulicz WC (2004). Microarray profiling of progesterone-regulated endometrial genes during the rhesus monkey secretory phase. Reprod Biol Endocrinol.

[B65] Mirkin S, Nikas G, Hsiu JG, Díaz J, Oehninger S (2004). Gene expression profiles and structural/functional features of the peri-implantation endometrium in natural and gonadotropin-stimulated cycles. J Clin Endocr Metab.

[B66] Okulicz WC, Ace CI, Franz C (2005). Temporal regulation of endometrial genes during inadequate secretory phases in the Rhesus monkey. Biol Reprod [special issue].

[B67] Torres MST, Ace CI, Okulicz WC (2002). Assessment and application of laser microdissection for analysis of gene expression in the Rhesus monkey endometrium. Biol Reprod.

[B68] Okulicz WC, Ace CI, Torres MS (2003). Gene expression in the rhesus monkey endometrium: differential display and laser capture microdissection. Front Biosci.

[B69] Abu JI, Konje JC (2000). Leukotrienes in gynaecology: the hypothetical value of anti-leukotriene therapy in dysmenorrhoea and endometriosis. Hum Reprod Update.

[B70] Malathy PV, Cheng HC, Dey SK (1986). Production of leukotrienes and prostaglandins in the rat uterus during peri-implantation period. Prostaglandins.

